# Physics-Informed Neural Network Modeling of Inflating Dielectric Elastomer Tubes for Energy Harvesting Applications

**DOI:** 10.3390/polym17172329

**Published:** 2025-08-28

**Authors:** Mahdi Askari-Sedeh, Mohammadamin Faraji, Mohammadamin Baniardalan, Eunsoo Choi, Alireza Ostadrahimi, Mostafa Baghani

**Affiliations:** 1 School of Mechanical Engineering, College of Engineering, University of Tehran, Tehran 1439957131, Iran; 2 Mechanical Engineering Department, Faculty of Engineering, Bu-Ali Sina University, Hamedan 6517838695, Iran; 3 Department of Civil and Environmental Engineering, Hongik University, Seoul 04066, Republic of Korea

**Keywords:** physics-informed neural networks, dielectric elastomer actuators, electromechanical response, nonlinear deformation, energy harvesting, hyperelastic materials, PINN modeling, Mesh-Free methods

## Abstract

A physics-informed neural network (PINN) framework is developed to model the large deformation and coupled electromechanical response of dielectric elastomer tubes for energy harvesting. The system integrates incompressible neo-Hookean elasticity with radial electric loading and compressible gas inflation, leading to nonlinear equilibrium equations with deformation-dependent boundary conditions. By embedding the governing equations and boundary conditions directly into its loss function, the PINN enables accurate, mesh-free solutions without requiring labeled data. It captures realistic pressure–volume interactions that are difficult to address analytically or through conventional numerical methods. The results show that internal volume increases by over 290% during inflation at higher reference pressures, with residual stretch after deflation reaching 9.6 times the undeformed volume. The axial force, initially tensile, becomes compressive at high voltages and pressures due to electromechanical loading and geometric constraints. Harvested energy increases strongly with pressure, while voltage contributes meaningfully only beyond a critical threshold. To ensure stable training across coupled stages, the network is optimized using the Optuna algorithm. Overall, the proposed framework offers a robust and flexible tool for predictive modeling and design of soft energy harvesters.

## 1. Introduction

The ongoing search for sustainable energy has increased efforts to develop new technologies that efficiently convert ambient mechanical energy into electrical power. Among these emerging technologies, dielectric elastomer generators (DEGs) have demonstrated substantial promise due to their ability to harness environmental stimuli and mechanical pressures for energy harvesting [1,2,3]. Compared to electromagnetic and piezoelectric harvesters, DEGs can theoretically achieve 80–90% energy-conversion efficiency and specific energies up to 1.7 J g^−1^, representing a tenfold increase in gravimetric energy density [3]. In particular, inflatable dielectric elastomer tubes, characterized by their lightweight, scalable, and highly deformable structures, stand out as especially attractive candidates for applications such as wave and flow energy conversion [4,5,6,7]. While most DEG studies have examined spherical balloon configurations, the cylindrical form has received comparatively little attention. This geometry offers practical advantages, such as easier application of controlled axial or radial forces and straightforward integration into experimental setups, making it a relevant and underexplored platform for coupled electromechanical analysis [8].

Despite their practical appeal, modeling the behavior of dielectric elastomer tubes undergoing large inflation remains particularly challenging due to the inherent nonlinearities and strong electromechanical coupling present in these systems [9,10,11]. The elastomeric materials commonly employed, such as silicone and acrylic elastomers, exhibit hyperviscoelastic behavior and are nearly incompressible, often requiring sophisticated constitutive models such as neo-Hookean elasticity. The large strains induced during inflation, coupled with voltage-driven Maxwell stresses, result in complex interactions between mechanical deformation and electric fields [12]. These interactions can even lead to instabilities, such as electromechanical thinning beyond critical voltages, further complicating predictive modeling and system optimization [13,14].

Historically, both analytical and numerical techniques have been employed to analyze dielectric elastomer actuators and generators [15,16,17,18]. Analytical approaches offer valuable insights into simplified, idealized scenarios, yet fall short when faced with the intricate deformation-dependent boundary conditions and nonlinear electromechanical feedback prevalent in practical systems. Conversely, numerical methods, particularly finite element modeling (FEM), have emerged as a robust means for handling these complexities. FEM allows detailed representation of realistic geometries and material anisotropy, including enhancements like fiber reinforcement, but requires substantial computational effort, careful mesh discretization, and intricate algorithmic strategies to ensure convergence and stability under coupled nonlinear conditions [19,20].

The computational challenges associated with FEM become especially significant in simulations involving inflating tube configurations. As the elastomer undergoes substantial volumetric expansion, the finite element mesh experiences severe distortion. Such distortion can reduce solution accuracy or even cause simulations to terminate prematurely [21]. To handle these large deformations, advanced yet computationally demanding methods, such as Arbitrary Lagrangian–Eulerian (ALE) formulations or frequent re-meshing techniques, are often necessary [22].

Recently, Physics-Informed Neural Networks (PINNs) have emerged as an innovative computational framework that elegantly combines the versatility of machine learning with the rigorous enforcement of physical laws [23,24,25]. PINNs differ fundamentally from traditional data-driven neural networks by embedding governing partial differential equations (PDEs), boundary conditions, and constraints directly into their optimization loss functions [26,27,28]. This approach eliminates the dependence on extensive labeled datasets, leveraging instead the underlying physics to guide solution accuracy and generalization. Consequently, PINNs provide a flexible yet rigorous alternative for solving coupled electromechanical problems characterized by strong nonlinearities and deformation-dependent boundary conditions [29].

The unique architecture of PINNs makes them particularly effective in addressing the distinct challenges posed by inflating DEGs. Their mesh-free nature entirely circumvents the mesh distortion issues that typically affect FEM in large-strain scenarios [20]. More importantly, by formulating a single comprehensive loss function that integrates all governing physics, PINNs provide an elegant and robust mechanism to enforce the strong, nonlinear coupling between electric and mechanical fields [30,31,32]. This unified approach eliminates the complexities associated with the staggered or monolithic solution schemes commonly employed in FEM, while also adeptly handling intricate and dynamically evolving boundary conditions.

While PINNs provide a mesh-free alternative, the proposed framework embeds the adiabatic gas relation and the coupled electromechanical field equations directly into the loss, thereby handling deformation-dependent boundary conditions without custom iterative coupling and sidestepping element distortion issues inherent to mesh-based discretizations in inflating DEGs [33,34,35].

In this paper, we introduce a novel Physics-Informed Neural Network framework specifically tailored to model the large-deformation electromechanical response of inflating dielectric elastomer tubes for sustainable energy harvesting. To our knowledge, this work represents a novel application of PINN-based modeling to dielectric elastomer generators under simultaneous gas-driven inflation and electrical loading. It addresses the critical challenge of deformation-dependent boundary conditions (e.g., adiabatic gas relations), which are generally intractable for analytical solutions and in FEM require iterative coupling schemes that add computational cost, whereas the proposed PINN framework is meshless and naturally enforces such conditions within its training process. Our proposed framework integrates incompressible neo-Hookean hyperelasticity, nonlinear electrostatic loading governed by Gauss’s law, and deformation-dependent boundary conditions directly into the network’s training process. By doing so, the PINN inherently captures the intricate coupling between mechanical deformation, electrical fields, and internal gas pressures—relationships that pose substantial analytical and numerical challenges. Being meshless, the approach can also be applied in situations with limited or incomplete data, enabling solutions for problems that are impractical for conventional analytical or finite element methods. We demonstrate that this approach effectively predicts realistic nonlinear responses such as pressure-induced stiffening at large inflations and electromechanical instabilities, surpassing the predictive capabilities of simpler analytical and traditional finite element methods [36]. Thus, our work establishes a powerful, flexible, and computationally efficient method for the predictive design and optimization of dielectric elastomer tube-based energy harvesters, further advancing the potential of this promising technology toward practical sustainability applications.

## 2. Materials and Methods

**Figure 1:**
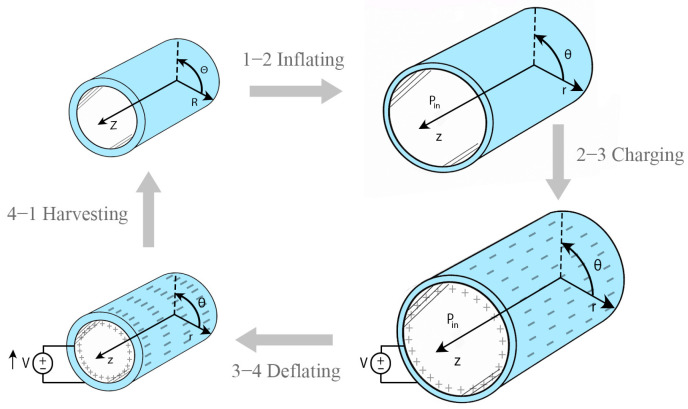
Conceptual schematic of the cyclic operation of a dielectric elastomer generator (DEG) tube.

For a general overview, the cyclic operation of the DEG tube is shown in Figure 1. It involves four stages: (1) undeformed initial state, (2) pneumatic inflation, (3) electrical charging of the inflated tube, and (4) deflation with partial recovery of mechanical work as electrical energy. This schematic serves as a conceptual introduction, with the detailed geometry and modeling described next.

**Figure 2:**
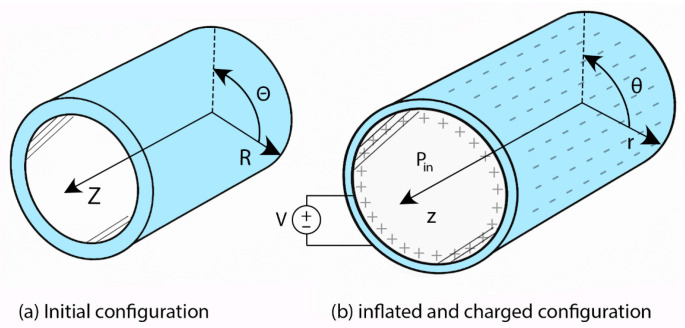
Geometry of the dielectric elastomer tube with radial electric field and internal inflation pressure.

This study considers a cylindrical dielectric elastomer actuator made of VHB 4910 [37], modeled as an incompressible neo-Hookean material, and subjected to radial electromechanical loading as illustrated in Figure 2. The incompressible neo-Hookean formulation is a fundamental hyperelastic model that captures the nonlinear elastic response of rubber-like solids undergoing large deformations. It assumes volume preservation during deformation and characterizes the material behavior through the shear modulus, enabling an accurate yet analytically tractable representation of isotropic elastomers. This choice is supported by its established use as a baseline for VHB 4910 in both computational and experimental dielectric elastomer studies (e.g., Diaz-Calleja et al. [38]; Pulok and Chakravarty [39]), where it reproduces the dominant nonlinear elastic trends over the moderate stretch range relevant to this work. Its analytical simplicity facilitates direct verification of the PINN predictions and ensures that the observed behaviors originate from the coupled electromechanical physics and boundary conditions rather than from complex constitutive fitting.

VHB 4910 (3M Company, Maplewood, MN, USA) is a commercially available acrylic elastomer, specifically a polyacrylate-based dielectric polymer tape, widely used in electromechanical applications due to its excellent hyperviscoelasticity and dielectric properties. It belongs to the class of soft, viscoelastic polymers with a crosslinked amorphous network structure that allows for large reversible deformations under mechanical and electrical loading. Physically, it exhibits near-incompressible behavior (Poisson’s ratio ≈0.49), a density of approximately 960 kg/m^3^, a glass transition temperature of around −30 °C, and a dielectric breakdown strength exceeding 25 kV/mm, making it suitable for energy harvesting in inflatable tube configurations [40]. Mechanically, it is modeled here as an incompressible neo-Hookean material with the parameters listed in Table 1. While VHB 4910 is inherently time-dependent, viscoelastic effects are not considered in this study, since the focus is on demonstrating the PINN framework for coupled electromechanical behavior with deformation-dependent boundary conditions and on evaluating the harvested and deformation-induced energies demanded in the cycle. This simplification, consistent with prior DEG studies employing hyperelastic models [39,41], ensures that the observed responses stem directly from electromechanical coupling rather than from rate-dependent effects.

In the framework, a four-stage process is implicitly used to model cyclic energy harvesting in dielectric elastomers. The elastomer tube starts out in a reference configuration that is undeformed and uncharged and then becomes mechanically inflated. When it is inflated, a voltage is applied across its wall, which causes electromechanical coupling and creates Maxwell stress in the radial direction as illustrated in Figure 2. This setup, which is now both inflated and charged, is the cycle’s peak-energy state. In the next phase, the system deflates while still being charged, and this is when electrical energy is collected. This harvesting phase takes advantage of the drop in capacitance while keeping the stored charge, which raises the voltage and gets rid of net energy. The amount of harvested energy over the cycle is expressed as follows:

(1) $$E_{\mathrm{harvest}}=\frac{1}{2}Q^{2}\left(\frac{1}{C_{4}}-\frac{1}{C_{3}}\right)$$

Equation (1) shows that the change in capacitance depends on the shape of the dielectric energy harvester is what controls energy recovery when the charge is fixed. A schematic of the problem geometry is shown in Figure 2. Let R and r be the radial coordinates in the reference and current configurations, respectively. Under the assumption of axisymmetric and plane strain, the deformation gradient tensor F in cylindrical coordinates is

(2) $$F=diag\left(\frac{dr\left(R\right)}{dR},\frac{r\left(R\right)}{R},1\right)$$

The incompressibility condition imposes $det\left(F\right)=1$, which preserves the volume during deformations. The right Cauchy–Green deformation tensor $C=F^{T}F$ is

(3) $$\;C=diag\left({\left(\frac{dr\left(R\right)}{dR}\right)}^{2},{\left(\frac{r\left(R\right)}{R}\right)}^{2},1\right)$$

and the first invariant of strain is

(4) $$I_{1}=tr\left(C\right)={\left(\frac{dr}{dR}\right)}^{2}+{\left(\frac{r\left(R\right)}{R}\right)}^{2}+1$$

A voltage V is applied between the inner and outer surfaces of the tube. This gives rise to a radial electric field in the current (deformed) configuration, derived from electrostatics as [42]

(5) $$E\left(r\right)=\frac{V}{\ln\left(\frac{r_{o}\left(R\right)}{r_{i}\left(R\right)}\right)}\cdot \frac{1}{r\left(R\right)}\;\hat{r}$$

where $r_{i}$ and $r_{o}$ are the deformed inner and outer radii, and $\hat{r}$ is the radial unit vector. This expression assumes the absence of free charges and radial symmetry in the field. The coupling between deformation and electric field is captured via the electromechanical enthalpy density $H_{\mathrm{eme}}$ defined as [15]

(6) $$H_{\mathrm{eme}}=W_{s}\left(C\right)-\frac{1}{2}\varepsilon_{0}\varepsilon_{r}J\;C^{-1}:\left(E⊗E\right),$$

where $W_{s}\left(C\right)$ denotes the elastic strain energy density, $\varepsilon_{0}$ is the vacuum permittivity, and $\varepsilon_{r}$ is the relative permittivity of the material. The corresponding total Cauchy stress tensor, incorporating incompressibility, elastic restoring forces, and both vacuum and material dielectric contributions, is expressed as [15]

(7) $$\sigma =-p\;I+\frac{\partial W_{s}}{\partial F}\cdot F^{⊤}+\varepsilon_{0}\left(E⊗E-\frac{1}{2}\left(E\cdot E\right)\;I\right)+\varepsilon_{0}\left(\left(\chi \cdot E\right)⊗E-\frac{1}{2}\left(E\cdot \chi \cdot E\right)\;I\right)$$

where p indicates the pressure arising from incompressibility, and χ is the electric susceptibility tensor, which for isotropic materials reduces to $\chi =\left(\varepsilon_{r}-1\right)\;I$. To characterize the elastic contribution, the strain energy function of an incompressible neo-Hookean material is adopted,

(8) $$W_{s}\left(C\right)=\frac{\mu }{2}\left(I_{1}-3\right),$$

where μ denotes the shear modulus of the elastomer. By substituting the strain energy function from Equation (8) and electrical field of Equation (5) into the general Cauchy stress expression of Equation (7), and carrying out the necessary mathematical manipulations, the Cauchy stress components are derived as

(9) $$\sigma_{rr}=-p+\frac{\mu }{3}\cdot \frac{-2{r^{\prime }}^{2}R^{2}+r^{2}+R^{2}}{R^{2}{\left(\frac{r^{\prime }\;r}{R}\right)}^{2/3}}+\frac{1}{2}\varepsilon_{0}\varepsilon_{r}{\left(\frac{V}{\;r\ln\left(r_{max}/r_{min}\right)}\right)}^{2}$$

(10) $$\sigma_{\theta \theta }=-p+\frac{\mu }{3}\cdot \frac{-{r^{\prime }}^{2}R^{2}-R^{2}+2r^{2}}{R^{2}{\left(\frac{r^{\prime }\;r}{R}\right)}^{2/3}}-\frac{1}{2}\varepsilon_{0}\varepsilon_{r}{{\left(\frac{V}{\;r\ln\left(r_{max}/r_{min}\right)}\right)}^{2}}^{2}$$

(11) $$\sigma_{zz}=-p+\frac{\mu }{3}\cdot \frac{-2R^{2}+{r^{\prime }}^{2}R^{2}-r^{2}}{R^{2}{\left(\frac{r^{\prime }\;r}{R}\right)}^{2/3}}-\frac{1}{2}\varepsilon_{0}\varepsilon_{r}{{\left(\frac{V}{\;r\ln\left(r_{max}/r_{min}\right)}\right)}^{2}}^{2}$$

where the functional dependencies $p\left(R\right)$ and $r\left(R\right)$ are omitted for brevity, and the prime symbol (′) indicates differentiation with respect to R. The only nontrivial mechanical equilibrium in the absence of body forces due to the minimal inertia of VHB 4910 membrane [43], is the radial equilibrium. Expressed in terms of the Cauchy stresses and using R as the radial coordinate, the equilibrium equation takes the form

(12) $$\frac{d\sigma_{rr}\left(R\right)}{dR}+\frac{dr\left(R\right)}{dR}\frac{\sigma_{rr}\left(R\right)-\sigma_{\theta \theta }\left(R\right)}{R}=0$$

Substituting the Cauchy stress from Equations (9) and (10), into the radial equilibrium equation (Equation (12)) yields

(13) $$\begin{array}{c} -p^{\prime }+\frac{5\lambda^{3}\mu rr^{\prime \prime }}{9R{\left(\frac{\lambda rr^{\prime }}{R}\right)}^{8/3}}+\frac{5\lambda^{3}\mu r^{\prime 2}}{9R{\left(\frac{\lambda rr^{\prime }}{R}\right)}^{8/3}}+\frac{2\lambda \mu rr^{\prime 2}r^{\prime \prime }}{9R{\left(\frac{\lambda rr^{\prime }}{R}\right)}^{8/3}}-\frac{\lambda \mu r^{\prime 4}}{9R{\left(\frac{\lambda rr^{\prime }}{R}\right)}^{8/3}}-\frac{5\lambda^{3}\mu rr^{\prime }}{9R^{2}{\left(\frac{\lambda rr^{\prime }}{R}\right)}^{8/3}}\\ \;\;\;\;\;\;+\frac{10\lambda \mu rr^{\prime 3}}{9R^{2}{\left(\frac{\lambda rr^{\prime }}{R}\right)}^{8/3}}+\frac{5\lambda \mu r^{3}r^{\prime \prime }}{9R^{3}{\left(\frac{\lambda rr^{\prime }}{R}\right)}^{8/3}}-\frac{10\lambda \mu r^{2}r^{\prime 2}}{9R^{3}{\left(\frac{\lambda rr^{\prime }}{R}\right)}^{8/3}}+\frac{\lambda \mu r^{3}r^{\prime }}{9R^{4}{\left(\frac{\lambda rr^{\prime }}{R}\right)}^{8/3}}\\ \;\;\;\;\;\;+\frac{5\lambda \mu \tau^{2}r^{3}r^{\prime \prime }}{9R{\left(\frac{\lambda rr^{\prime }}{R}\right)}^{8/3}}-\frac{10\lambda \mu \tau^{2}r^{2}r^{\prime 2}}{9R{\left(\frac{\lambda rr^{\prime }}{R}\right)}^{8/3}}-\frac{5\lambda \mu \tau^{2}r^{3}r^{\prime }}{9R^{2}{\left(\frac{\lambda rr^{\prime }}{R}\right)}^{8/3}}-\frac{\varepsilon_{0}\varepsilon_{r}V^{2}}{r^{3}\ln^{2}\left(\frac{r_{\max}}{r_{\min}}\right)}\cdot r^{\prime }\\ \;\;\;\;\;\;+\frac{\varepsilon_{0}\varepsilon_{r}V^{2}}{r^{2}\ln^{2}\left(\frac{r_{\max}}{r_{\min}}\right)}=0 \end{array}$$

Equation (13) represents the final form of the radial equilibrium equation, obtained by incorporating the Cauchy stress components derived from the constitutive relations into the general equations of motion. In the present study, this coupled electromechanical relation is solved in conjunction with deformation-dependent boundary conditions using a physics-informed neural network framework, as detailed in Section 2.1. To make it easier to compare and generalize, the electric voltage, axial force, internal pressure and energy densities are made to have no dimensions as [44]

(14) $$\bar{V}=\frac{V}{H\sqrt{\mu /\varepsilon_{r}}},\;{\bar{F}}_{Z}=\frac{F_{Z}}{\mu H^{2}},\;\bar{P_{0}}=\frac{P_{0}}{\mu },\;{\bar{W}}_{s}=\frac{W_{s}}{\mu V_{0}}.$$

where H is the height and $V_{0}$ is reference volume of the dielectric tube.

### 2.1. PINN-Based Solution of the Coupled Electromechanical Problem

When modeling dielectric elastomer tubes for energy harvesting, dealing with boundary conditions is often an enormous challenge for analytical and semi-analytical methods. Classical solutions often use idealized boundary assumptions, like constant inflation pressure or perfectly traction-free surfaces, which do not fully show how real devices work. In actual systems, the inflation pressure is often connected to the deformed geometry through gas compressibility or pneumatic flow effects. This creates an implicit connection between the boundary loading and the unknown deformation field. To solve these types of problems analytically, iterative schemes or special approximations are usually needed. These can be hard to work with and may not work for more complicated boundary situations, like soft mechanical interfaces or pre-stretch that change with space. In this work, an axisymmetric tube with a spatially uniform but deformation-dependent inner pressure is considered, and the manner in which this coupling is imposed in the PINN is described.

To address these modeling challenges and capture the nonlinear coupling between deformation, electromechanical loading, and boundary conditions, a physics-informed neural network (PINN) framework is developed. The framework employs two subnetworks that are trained simultaneously to predict the deformed radius $r\left(R\right)$ and the pressure field $p\left(R\right)$. The Cauchy traction $\sigma_{rr}$ on the inner and outer surfaces is not an independent network output; it is computed deterministically from r(R) and p(R) via the hyperelastic and Maxwell stress constitutive relation of Equation (9). Consequently, any candidate fields proposed by the network determine the boundary tractions, and the loss penalizes any mismatch with the boundary data. This directly constrains the network’s guesses to satisfy the boundary physics through the constitutive relations. Both subnetworks are coupled through a shared physics-informed loss function that enforces equilibrium equations, the incompressibility constraint, and boundary conditions, ensuring consistency between the predicted mechanical fields

(15) $$L_{total}=L_{PDE}+\alpha L_{inc}+\beta L_{BC}$$

where α and β are weighting coefficients. The term $L_{PDE}$, computed over $N_{f}$ collocation points, penalizes the residual of the radial equilibrium equation, which incorporates both the elastic and electromechanical stress contributions under the combined action of inflation pressure and applied electric field

(16) $$L_{PDE}=\frac{1}{N_{f}}\sum_{i=1}^{N_{f}}{\left[Residual\;of\;Equation\;(13)\;at\;R_{i}\right]}^{2}$$

The incompressibility constraint is imposed over $N_{f}$ collocation points as

(17) $$L_{inc}=\frac{1}{N_{f}}\sum_{i=1}^{N_{f}}{\left[r^{\prime }\left(R_{i}\right)\cdot \frac{r\left(R_{i}\right)}{R_{i}}-1\right]}^{2}$$

The boundary condition term $L_{BC}$, enforces a uniform but deformation dependent inner pressure and a traction free outer surface by comparing the constitutively implied normal traction $\sigma_{rr}$, computed from the current network predictions r(R) and p(R), with the boundary data at $N_{b}$ boundary collocation points on ${R=R}_{i}$ and ${R=R}_{o}$; the inner pressure $P_{\mathrm{in}}(r)$ is recomputed from the current volume at each pass and the resulting mismatch is penalized, through

(18) $$L_{BC}=\frac{1}{N_{b}}\sum_{j=1}^{N_{b}}\left({\left[\sigma_{rr}\left(R_{j}\right)-P_{\mathrm{in}}\right]}^{2}\;\mathrm{if}\;R_{j}\in R_{i},\;{\left[\sigma_{rr}\left(R_{j}\right)\right]}^{2}\;\mathrm{if}\;R_{j}\in R_{o}\right)$$

In this study, the applied inflation pressure $P_{\mathrm{in}}(r)$ reflects the behavior of a compressible gas (air) used to inflate the dielectric elastomer tube, modeled as [45]

(19) $$P_{\mathrm{in}}(r)=P_{0}{\left(\frac{V_{0}}{V\left(r\right)}\right)}^{\gamma }$$

where $P_{0}$ is the reference pressure corresponding to initial volume $V_{0}$, V is the current internal volume of the deformed tube, and γ is the polytropic index (equal to 1 for isothermal processes, or approximately 1.4 for adiabatic compression of air). In this work, the inflation process is considered to be adiabatic because it happens rapidly and VHB 4910 is a poor heat conductor [46]. This deformation-dependent pressure–volume relation ($P_{\mathrm{in}}$) creates a direct coupling between the boundary loading and the unknown deformation field, which is challenging to impose in analytical solutions and typically requires iterative updates of the applied pressure in finite element models. In the proposed PINN framework, this coupling is incorporated directly into the boundary loss term ($L_{BC}$), allowing it to be satisfied automatically during training without iterative load adjustments.

All necessary spatial derivatives are provided by automatic differentiation, allowing the gradient to flow smoothly throughout the training process. By using the already defined constitutive framework, the governing quantities are directly taken from the predicted radius and pressure fields. This makes sure that the physical limits of the coupled electromechanical system are strictly followed throughout the computational domain.

**Figure 3:**
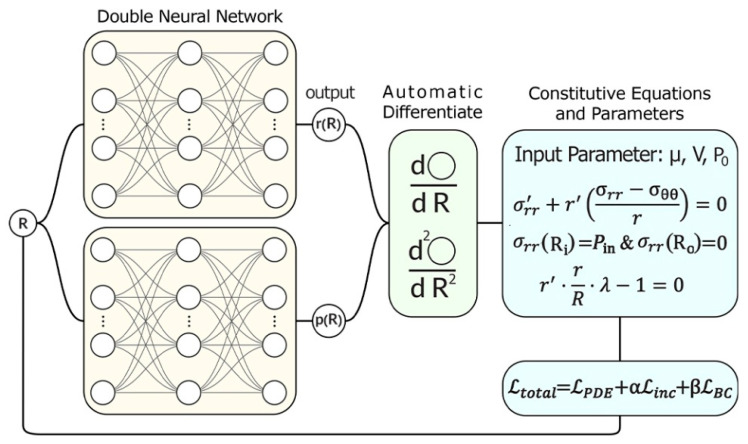
PINN architecture of the electromechanical inflation of dielectric tube.

The PINN model is meant to be a close match for the coupled electromechanical fields that control how the dielectric elastomer tube changes shape. Two neural networks are used: one predicts the deformed radial coordinate r(R), and the other predicts the hydrostatic pressure field p(R). The only variable input to the neural networks is the reference radial coordinate R. Other quantities such as the shear modulus (μ), dielectric constant $\varepsilon_{r}$, applied voltage (V), and reference pressure ($P_{0}$) are treated as fixed problem parameters. The output fields are then used to compute the mechanical stresses, electric field, and equilibrium residuals, as illustrated in Figure 3.

The network architecture is setup with enough depth and width to capture the highly nonlinear connection between the elastic deformation and the electric field that is applied, while still keeping convergence stable throughout training. The model learns electromechanically consistent field solutions directly from the equations by enforcing the governing physical laws through the loss formulation. It does not need labeled data or external discretization.

Since the electromechanical equilibrium is highly nonlinear and there are stiff constraints like incompressibility and set boundary conditions, a staged training strategy is used. There are a set number of epochs in each stage of the optimization process, and the model states are saved at each stage to help improve the solution. This helps the optimizer systematically deal with conflicting constraints and stops it from converging too soon to local minima.

Penalty weights α and β, which determine how much incompressibility and boundary condition losses matter in relation to each other, are treated as hyperparameters and optimized along with the learning rate and training stages. The Optuna framework is utilized to optimize these hyperparameters. It does a systematic search over ranges that are physically relevant. This PINN-based method is a flexible, mesh-free methods to model the strongly coupled electromechanical behavior of energy-harvesting dielectric elastomers. It is a powerful tool for the predictive design and analysis of these types of systems.

### 2.2. Optimization Framework: Optuna

Training a physics-informed neural network (PINN) for the inflating-tube problem requires balancing three loss channels—governing-equation residuals, incompressibility, and deformation-dependent boundary conditions—whose magnitudes differ by several orders. Manually guessing penalty weights or learning-rate schedules often leads to unstable optimization and sub-optimal accuracy. We therefore adopted Optuna, an open-source automatic hyperparameter optimization (HPO) library that combines sophisticated samplers, asynchronous scheduling and built-in pruning to search high-dimensional spaces efficiently Akiba et al. [47]. As for the search space, five hyper-parameters were tuned:

α (Incompressibility Penalty Coefficient): This coefficient (α in Equation (15)) regulates the severity of the penalty given to violations of the incompressibility constraint (J=1). Larger values can influence convergence if too dominant, but they also enforce incompressibility more strictly.

β (Boundary Condition Penalty Coefficient): In the overall loss, this coefficient (β in Equation (15)) governs the relative weight of fulfilling the traction-free boundary conditions ($\sigma_{rr}=0$ at inner and outer surfaces).

$\eta_{0}$ (Initial Learning Rate): The Adam optimizer’s initial learning rate is one that undergoes exponential decay during training.

All remaining network hyperparameters (eight hidden layers, sine activation, Glorot uniform weight initialization) were fixed to confine the search. At the end of the last epoch, the three un-weighted components of the loss are recorded, $L_{PDE}^{final}$, $L_{inc}^{final}$, $L_{BC}^{final}$, and the scalar objective passed back to the study is

(20) $$Objective=L_{PDE}^{final}+L_{inc}^{final}+L_{BC}^{final}$$

The study employs a Tree-of-Parzen Estimator sampler with 100 trials and a median pruner to halt unpromising runs early. After optimization, the best parameter set is retrained to produce the final results. These implementation choices—including a compact yet expressive network architecture, gradient-exact training, and a targeted hyperparameter search—collectively establish a robust, mesh-free framework for modeling large extension–torsion deformations in incompressible hyperelastic cylinders.

## 3. Results and Discussion

The trained physics-informed neural network (PINN) provides us spatially resolved fields for the deformed radius r(R), hydrostatic pressure p(R), and derived electromechanical quantities like stress components and electric field intensity. All of the simulations are performed on a cylindrical dielectric elastomer tube made of VHB 4910, which is modeled as an incompressible neo-Hookean solid. Table 1 shows the material parameters that were utilized in the simulations.

To model the entire energy harvesting cycle, a staged training approach was used. Transfer learning was used in an approach which made sense in the real world: an initial PINN model was trained to learn the purely mechanical inflation process from a configuration that was not deformed (state 1) to a pre-inflated shape (state 2). By using this mechanical solution, the training for the charging process (from state 2 to state 3) is started, during which a constant voltage was applied across the expanded tube. After that, the model from the charged configuration (state 3) was adjusted to show the deflation process (state 3 to state 2), but this time with the total charge kept the same. The stored charge stays the same, so the voltage in the tube goes up during deflation because the capacitance goes down. Throughout this staged cycle (inflation, charging, and deflation), the internal pressure boundary condition was imposed through the deformation-dependent adiabatic pressure–volume relation (Equation (19)). For completeness, a separate comparison between constant-pressure and adiabatic inflation was performed to demonstrate the limitations of fixed-pressure assumptions, but all cycle simulations reported here use the adiabatic formulation.

This progressive training sequence makes it easier to converge when fields are strongly coupled and makes it easier to solve non-monotonic deformation paths quickly. It also shows the order of events in experiments and energy harvesting cycles, which makes sure that history-dependent effects are modeled in a realistic way.

**Figure 4:**
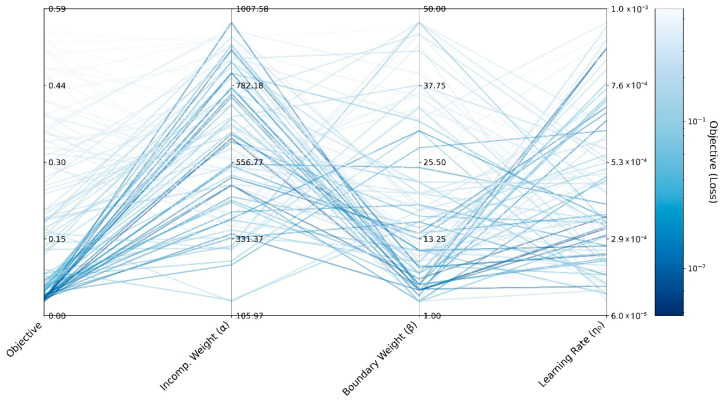
Parallel coordinates plot of Optuna hyperparameter trials, colored by final loss (log scale).

To ensure robust convergence across all loading stages, hyperparameter optimization was used on a representative hybrid configuration that included both mechanical and electrical contributions. In particular, a mix of states 2 and 3 was used to show how pressure-induced deformation and voltage-driven electromechanical coupling work together. The Optuna framework Akiba, Sano, Yanase, Ohta and Koyama [47] was employed to tune initial learning rate, the incompressibility and boundary condition penalty weights (α and β), and the network architecture. Optimizing this hybrid setup made training more stable and made sure that constraints were enforced correctly throughout the whole cycle. Figure 4 visualizes the hyperparameter search space and how it affects model performance. Low-loss trials are shown in color gradients in a parallel coordinates plot.

Among all 100 trials, the optimization process found the best set of hyperparameters for an objective value of 0.004, with α=633.2, β=3.1, and $\eta_{0}=3.07\times 10^{-4}$. This optimal configuration was adopted in all subsequent model trainings.

**Figure 5:**
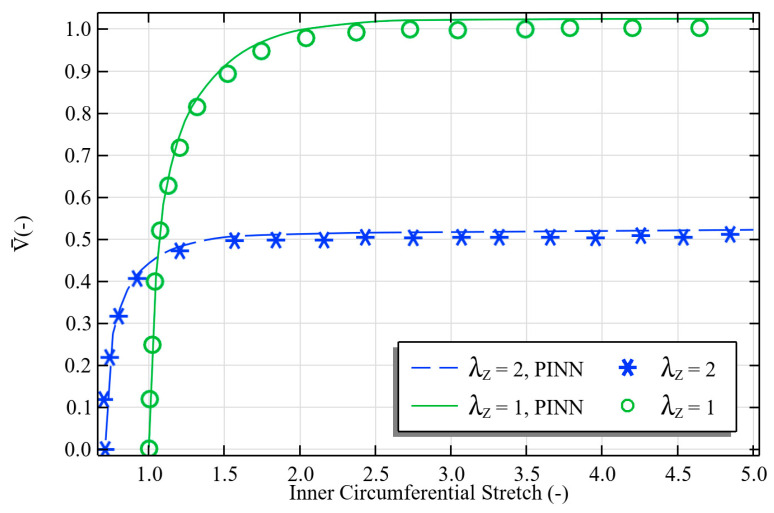
PINN accurately predicts voltage–stretch behavior in tubular dielectric elastomers, showing excellent agreement with reference data. Adapted from [44], Elsevier, 2017.

In terms of predictive performance and validating against previous research [44], Figure 5 shows the precision of the proposed PINN in modeling the relationship between voltage and stretch for tubular dielectric elastomers with different levels of axial pre-stretch ($\lambda_{Z}=1$ and $\lambda_{Z}=2$). The PINN accurately captures the nonlinear electromechanical response, proving that it works well for energy harvesting applications where it is important to know exactly how voltage and deformation are coupled. The model also captures the turning point behavior at higher pre-stretch, which is linked to the start of mechanical instability. However, this is not the main focus; the main focus is on modeling energy harvesting efficiency and dynamic behavior.

**Figure 6:**
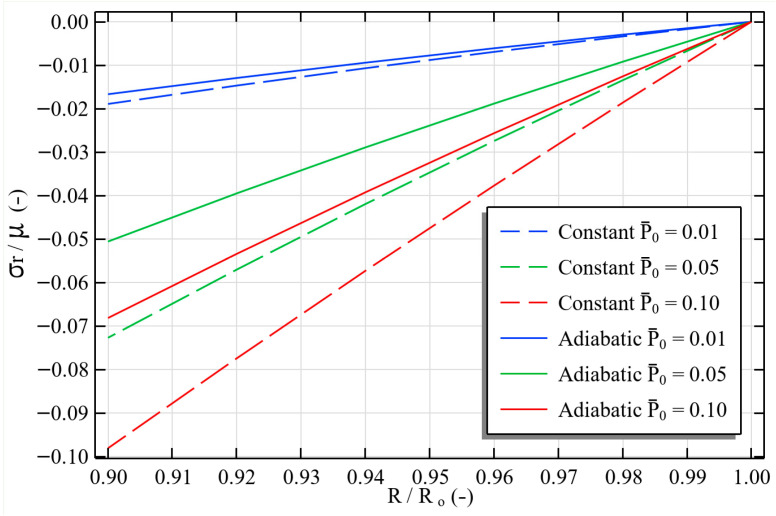
Radial stress distribution across the tube thickness for constant pressure (dashed lines) and adiabatic inflation (solid lines) scenarios at three normalized reference pressures.

The radial stress distribution across the tube thickness was evaluated for both constant-pressure and adiabatic inflation scenarios at three normalized reference pressures (${\bar{P}}_{0}=0.01,0.05,0.10$), where $\bar{P_{0}}=P_{0}/\mu$, as illustrated in Figure 6. In the constant-pressure case (dashed lines), the applied load remains fixed during deformation, producing higher radial stresses and greater circumferential stretch. In contrast, the adiabatic case (solid lines) exhibits a marked reduction in internal pressure during inflation due to the pressure–volume coupling of the enclosed gas. For the highest load case ($\bar{P_{0}}=0.1$), this coupling lowers the attained internal pressure by approximately 30.5%, reducing the maximum circumferential stretch from 2.037 to 1.32, corresponding to a 35% decrease in deformation capability. Physically, this reflects the inability of a rapidly expanded gas to maintain its initial pressure under adiabatic conditions, thereby limiting the mechanical response. Such sensitivity to the boundary-condition model underscores the importance of incorporating physically realistic loading scenarios when predicting the performance of dielectric elastomer systems [48,49]. The proposed PINN framework naturally accommodates these deformation-dependent boundary conditions within its boundary loss formulation, enabling accurate simulation without iterative BC updates or remeshing.

**Figure 7:**
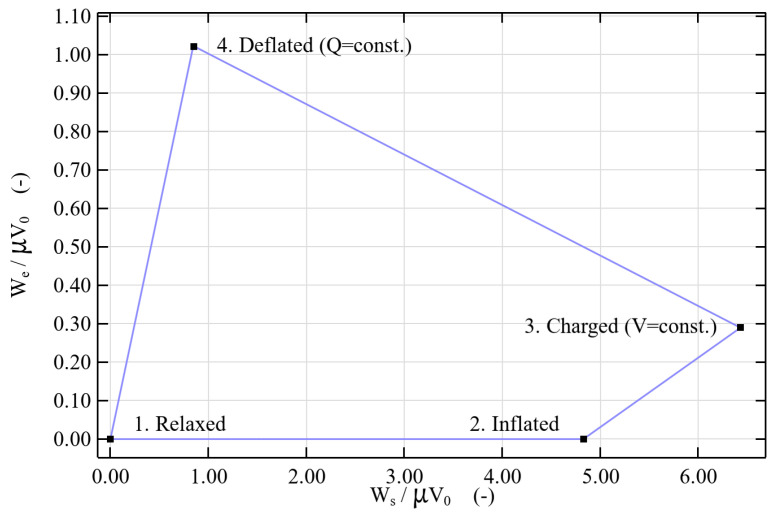
PINN-predicted dimensionless electromechanical cycle of a VHB 4910 tube at $\bar{P_{0}}=0.3$ and $\overset{¯}{V}=32$.

To investigate the complete harvesting energy cycle with the dielectric tube, PINN is trained for different state as illustrated in Figure 7 per $\bar{P_{0}}=0.3$ and $\bar{V}=32$. The tube starts out relaxed and deformation-free (1). Then, air pressure (2) inflates it quasi-statically, and a constant voltage (3) is applied. This causes Maxwell stress to increase the mechanical and electrical energy. The system then deflates at a constant charge (4). During the process, the mechanical energy goes down and transfer to the electrical energy, hence the voltage goes up as the capacitance goes down (preservation of the stored charge). It is important to note that the mechanical energy does not go back to zero at the end of the cycle, which means that Maxwell stress is still present at (4). The process ends with a return to the original state after discharge.

**Figure 8:**
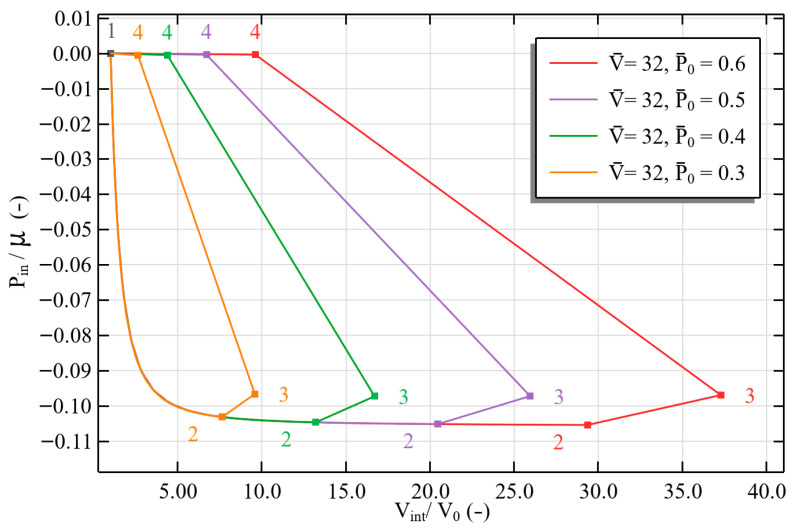
PINN-predicted pressure–volume curves at fixed $\bar{V}=3.2$ for increasing $\bar{P_{0}}=0.3$ in the harvesting cycle.

For different reference pressures $\bar{P_{0}}$ and a fixed voltage $\bar{V}=32$, Figure 8 shows the normalized internal pressure versus normalized internal volume. The internal volume increases dramatically as $\bar{P_{0}}$ increases, the internal volume rises significantly—up to a 290% increase from 0.3 to 0.6 of inflation—indicating more efficient inflation. This is because the tube wall is thinned by the initial mechanical pressurization, which improves the tube’s electromechanical responsiveness, it is to be noted that as fixed voltage charging occurs the pressure increases due to the superposition of maxwell and mechanical inflating pressure. The curves also show a sharp volumetric expansion with minimal pressure change at higher $\bar{P_{0}}$. This is indicative of a limit-point instability, also known as ballooning instability, that is seen in thin-walled hyperelastic tubes [50]. The residual stretch due to the maxwell stresses increases with $\bar{P_{0}}$ after deflation completes: the internal volume increases by more than 265% to 9.61 of its undeformed value at highest $\bar{P_{0}}=0.6$, from 2.63 at $\bar{P_{0}}=0.3$. These findings highlight how important inflation pressure is for permitting significant, harvestable volume changes while operating at a fixed voltage.

**Figure 9:**
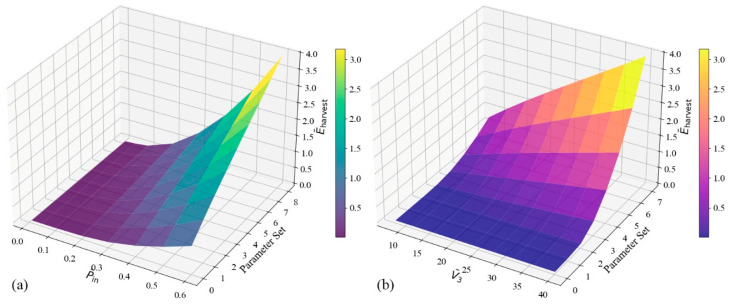
Predicted harvested energy surfaces from PINN: variation with nondimensional pressure (**a**) and voltage (**b**) across parameter sets.

For a specific dielectric tube energy harvester configuration, Figure 9 illustrates the relationship between harvested energy and nondimensional pressure and voltage across a range of parameter sets, each of which represents a unique combination of these two variables.

Harvested energy improves nonlinearly with pressure in Figure 9a, especially for higher parameter sets. This pattern implies that mechanical loading significantly boosts energy output, most likely as a result of the dielectric structure deforming more under pressure. As pressure increases, the mechanical contribution becomes more significant, emphasizing how crucial mechanical pre-strain is to allowing for significant energy gains. This suggests that pressure plays a significant role in performance, particularly in systems built to endure and benefit from high mechanical strains.

Figure 9b, on the other hand, displays harvested energy as a function of applied voltage. In contrast to the pressure case, energy output improves more gradually even though it increases steadily with voltage. Interestingly, harvested energy is essentially constant across parameter sets in the low-voltage region. This indicates a threshold behavior below which energy harvesting is negligible and implies that small voltages are insufficient to produce significant electromechanical effects. Beyond this point, energy rises more precipitously, indicating the electric field’s increasing role in causing polarization and deformation.

The plots show that energy harvesting is improved by both pressure and voltage, but that pressure has a stronger and nonlinear effect, especially at high parameter levels. In the meantime, for voltage to work, it needs to rise above a specific activation level. These findings highlight how crucial system-level design is for optimizing harvesting efficiency in dielectric tube systems by balancing mechanical loading, electrical input, geometry, and material properties.

**Figure 10:**
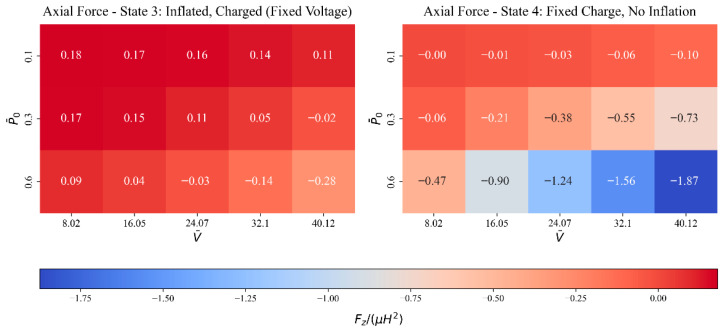
Axial force heat maps for State 3 and State 4.

Axial force heat maps at two critical points in the energy harvesting cycle are shown in Figure 10. As expected from internal pressure and constrained axial deformation, the axial force is primarily tensile in State 3, where the tube is both inflated and charged at a fixed voltage. It may appear counterintuitive given the active inflation, but the axial force turns compressive at higher voltages and pressures.

This arises from the interaction between radial electric compression and circumferential expansion induced by Maxwell stress. Under the constraints of incompressibility and plane strain, the material cannot stretch axially. Instead, it builds up axial compressive stress to keep its volume while allowing hoop and radial deformation. In State 4, where inflation is removed but charge is conserved, this effect is even stronger: the rising voltage makes Maxwell stress even stronger, which causes a lot of axial compression. These results show how important electromechanical coupling and stress redistribution driven by constraints are in soft dielectric systems

## 4. Conclusions

This study showed how to use a physics-informed neural network (PINN) to model the nonlinear electromechanical behavior of a VHB 4910 dielectric elastomer tube as it inflates, charges with electricity, and then deflates. By embedding the governing physics directly into the learning process, the framework accurately captures the critical stages of the energy harvesting cycle, enabling precise predictions of stress, deformation, and field distributions under realistic, deformation-dependent boundary conditions.

The PINN framework is not confined to VHB 4910 and can be applied to other dielectric elastomers, like silicone or polyurethane-based polymers, by incorporating their specific hyperelastic models (e.g., Mooney–Rivlin or Ogden) and dielectric properties into the governing equations. In addition, the framework can be readily extended to account for more complex behaviors such as viscoelasticity, enabling the study of rate-dependent effects that are important for long-term performance and realistic operating conditions. This flexibility makes the model a useful tool for studying various soft polymer materials for energy harvesting.

The model predicted that at higher pressure levels, the internal volume increases by more than 290% during inflation, with a residual volume after deflation of more than 9.6 times the undeformed value, indicating a significant potential for energy harvesting. The accuracy of the electromechanical predictions was confirmed by the voltage-stretch response, which closely matched reference data. Furthermore, the analysis of axial forces revealed a transition from tension to compression at higher voltages and during deflation. This phenomenon, driven by Maxwell stress under the constraint of incompressibility, represents an important and often overlooked aspect of soft dielectric mechanics.

The findings underscore a clear design paradigm for these energy harvesters: mechanical pressure is the primary driver of potential energy output, while applied voltage acts as a critical enabler that must surpass a specific threshold to initiate efficient harvesting. This work establishes the PINN methodology as a powerful, flexible, and computationally efficient tool for the predictive design and optimization of soft energy systems.

Future work will extend this framework to incorporate more complex material behaviors such as viscoelasticity, enabling the study of rate-dependent effects that are important for long-term performance and realistic operating conditions. Moreover, the inherent differentiability of the neural network solution, together with systematic experimental validation, paves the way for inverse design and optimization, where experimental data can be directly leveraged to identify material parameters and guide the automated discovery of novel properties, geometries, and loading protocols to maximize energy harvesting efficiency for practical sustainability applications.

## Figures and Tables

**Table 1:** Material parameters used for the VHB 4910 dielectric elastomer [8].

Property	Symbol	Value
Shear modulus	μ	73 kPa
Relative permittivity	$\varepsilon_{r}$	4.68
Vacuum permittivity	$\varepsilon_{0}$	$8.854\times 10^{-12}\;F/m$
Adiabatic index (air)	γ	1.4

## Data Availability

The data presented in this study are available on request from the corresponding author. The data are not publicly available due to privacy concerns.

## Abbreviations

The following abbreviations are used in this manuscript:

PINN	Physics-Informed Neural Network
DEG	Dielectric Elastomer Generator
FEM	Finite Element Method
ALE	Arbitrary Lagrangian–Eulerian
PDE	Partial Differential Equation
HPO	Hyperparameter Optimization
VHB	Vinyl Hybrid Bonding (commercial elastomer name: VHB 4910)
